# On the Differential Diagnosis of Swellings in the Neck.—I

**Published:** 1894-12-15

**Authors:** Alexander Miles

**Affiliations:** Surgical Tutor Royal Infirmary, Edinburgh


### MEDICAL PROGRESS AND HOSPITAL CLINICS.

[*The Editor will be glad to receive offers of co-operation and contributions from members of the profession. All letters should be addressed to* THE EDITOR, THE LODGE, PORCHESTER SQUARE, LONDON, W.]

### ON THE DIFFERENTIAL DIAGNOSIS OF SWELLINGS IN THE NECK.

By ALEXANDER MILES, M.D., F.R.C.S. Edin., Surgical Tutor Royal Infirmary, Edinburgh.

### I.—FLUID SWELLINGS.

The differential diagnosis of swellings in the region of the neck is often a matter of considerable difficulty, partly on account of the great variety of morbid conditions which may be met with here, and partly from the anatomical arrangements. The cervical fascia being a particularly dense and complicated structure, causes new growths and collections of fluid under it, to follow directions and to assume positions which are only to be explained by accurate anatomical knowledge. The physical signs of these swellings also are often so masked by it that their true significance is obscured. The numerous large blood vessels, and the close relations which they must necessarily bear to morbid materials, in many cases lend to the latter appearances which are adventitious.

One of the first questions to be decided in the diagnosis of a swelling in the neck is that of its consistence. Is it fluid or solid?

Among the possibilities, if it be fluid, are (1) abscess; (2) cyst; (3) aneurism; (4) ranula; (5) enlarged bursa. As this is perhaps the order of frequency in which these conditions are met with, it may be convenient to consider them now in this order, although clinically it is often simpler to exclude first the less likely conditions.

To save repetition it may be stated here that any one of these fluid swellings by close contact with a large blood vessel may assume some of the signs of an aneurism by receiving a transmitted pulsation. This pulsation, however, is non-expansile, is unaccompanied by a bruit, and is not associated with any difference in the distal pulse on the two sides of the body. Aneurism occurs oftenest in males and in advanced life.

(1) **Abscess.**—An abscess in the neck has, of course, the clinical signs and symptoms of a similar condition elsewhere, namely, a certain degree of heat, discolouration, swelling, with fluctuation, pain and tenderness, as well as general constitutional disturbance in the shape of rigors, high temperature, gastro-intestinal derangements, and so on. These, together with the history of an antecedent inflammation, acute or chronic, will usually settle that it is an abscess; and the next point to determine is its source. (*a*) Undoubtedly the most common source of suppuration in the neck is the lymphatic glands, which are so numerous in this region, and which drain areas where local irritation of a septic or other nature is more than ordinarily prone to occur. The situation of the swelling, the history of its onset, and the discovery of some obvious source of irritation, such as carious teeth, inflamed tonsils, sore throat, eczema capitis, or chronic middle-ear disease, will lead to a correct diagnosis in a majority of cases, although sometimes no cause can be assigned. (*b*) Following on severe attacks of scarlet fever, measles, &c., in debilitated children, a diffuse cellulitis of the neck is not uncommon. It gives rise to great constitutional disturbance and most pronounced local signs. The swelling at first is firm and brawny, with much œdema around it, but soon softens and exhibits the characters of a wide-spread suppuration. A very serious, and too often fatal form of this condition, called "Ludwig's angina," occurs in the submaxillary region, but is not very common. (*c*) A retro-pharyngeal abscess, or collection of pus between the spinal column and the pharynx, not unfrequently shows as a swelling on one side, sometimes on both sides, of the neck. The diagnosis of this condition will be aided by finding some such cause for the abscess as spinal caries, inflammation in the pharynx or tonsils, or in rare cases chronic middle-ear suppuration. Attributable to this last cause the writer has recently seen a case in which a large swelling appeared on both sides of the neck. The other symptoms of retro-pharyngeal abscess are difficulty and pain on swallowing, snoring respiration, stiffness of the neck, and a soft fluctuating swelling in the back of the pharynx. (*d*) Occasionally an abscess starting in the mediastinal glands shows as a swelling above the sternum.

Having determined the nature of an abscess, the next point to decide is its relation to the cervical fascia. If superficial to this structure the swelling will be relatively localised, and the fluctuation distinct once pus has fairly formed. If under the pretracheal layer fluctuation is obscured, pressure symptoms more common, and the fluid may gravitate into the thoracic cavity. The retro-pharyngeal abscess is in the space between the prevertebral layer of the cervical fascia and the bucco-pharyngeal fascia which encloses the pharynx.

Abscesses have to be diagnosed from the other fluid swellings above named, and occasionally from a rapidly growing sarcoma, which is soft, but not fluctuating, and has a more or less irregular surface. To distinguish between acute and chronic abscess is, of course, a simple matter.

(2) **Cysts.**—Inasmuch as most of the cystic tumours occurring in the neck have their origin in some maldevelopment of one or other of the branchial clefts which occupy the neck of the young fœtus, it has been proposed to include them all under the term "Branchial Cysts." The greater number of them are congenital, a fact which materially aids in the differential diagnosis. They are oval, fluctuating, and unilateral, usually occurring on the left side and in the upper part of the neck. (*a*) The commonest variety is what is known at *hydrocoele of the neck*, and consists in a rounded smooth and elastic swelling, of a bluish colour, containing a clear serous fluid, saline and albuminous, situated along the line of the sterno-mastoid muscle. It is devoid of pain, and unless of great size gives rise to no discomfort. The skin over it is free, and unless when multiple it has no deep connections. (*b*) Dermoid cysts are also congenital as a rule. They result from the inclusion of a small piece of epiblast in the mesoblast of the developing fœtus, and as the cyst wall, therefore, contains sebaceous glands and hair follicles, the cavity is filled with sebaceous material and hair. As distinguishing them from hydrocoele of the neck, they are firmer, more deeply situated, and have thicker walls. When in the submaxillary region they are apt to be mistaken for ranulæ. (*c*) Sanguineous cysts are rare, and are either serous or dermoid cysts, into which a hæmorrhage has taken plaee, or with which a vein, usually the anterior jugular, has been in communication. Their colour is the only characteristic feature they possess till opened into. (*d*) Hydated cysts, of course, may occur here as elsewhere, but can only be detected by the microscope exhibiting hooklets in their contents. (*e*) A cyst may form in an accessory thyroid body, but is very difficult of diagnosis. All these cystic tumours are slow, but sometimes erratic, in their growth, increasing or diminishing without any obvious cause. They are to be diagnosed from one another, from the other fluid tumours above named, and from sarcomata and fatty tumours. The non-congenital forms may be confused with broncho-cele when in the region of the thyroid gland; and the congenital ones with spina bifida and meningo-cele when situated posteriorly.

(3) **Aneurisms** are less difficult to distinguish. Their anatomical position, their characteristically expansile pulsation, with a bruit on auscultation; the effect of proximal pressure on the size and pulsation of the swelling, and the implication of surrounding structures, if any, will indicate the diagnosis.

(4) **Ranulæ**, in addition to the swelling in the mouth, when of unusual size sometimes produce a fulness in the submaxillary region. They have to be distinguished from serous cysts (hydrocele of neck), and, from the condition to be next considered, enlarged hyoid bursa.

(5) **Enlarged Bursæ.**—Between the hyoid bone and the thyroid cartilage, on the surface of the thyro-hyoid membrane, is a bursa which sometimes becomes enlarged to a considerable extent. The writer not long ago operated on a case in which a swelling, simulating a goitre, appeared in the neck, coincidently with one in the mouth, which pushed the tongue against the roof of the month, seriously interfering with eating, breathing, and speaking. The diagnosis was only made certain after ascertaining its connections by incision.

